# Role of Supervisor Consultation Toward Work Engagement: A Prospective Cohort Study

**DOI:** 10.1016/j.shaw.2024.02.003

**Published:** 2024-02-09

**Authors:** Nuri P. Adi, Tomohisa Nagata, Kiminori Odagami, Masako Nagata, Koji Mori

**Affiliations:** 1 Department of Occupational Health Practice and Management, Institute of Industrial Ecological Sciences, University of Occupational and Environmental Health, Japan; 2 Department of Community Medicine, Faculty of Medicine, Universitas Indonesia, Jakarta, Indonesia; 3 Department of Occupational Medicine, School of Medicine, University of Occupational and Environmental Health, Japan

**Keywords:** Perceived supervisor support, Supervisor consultation, Work engagement

## Abstract

**Background:**

We examined the association between supervisor consultation, as an actual practice representing supervisor support, and work engagement.

**Methods:**

This was a prospective cohort study in Japan, involving 14,026 participants who met the requirement for a one-year follow-up. Supervisor consultation was measured using a single question, and work engagement was defined using the Japanese version of the nine-item Utrecht Work Engagement Scale (UWES-9). Associations were examined using linear regression analysis.

**Results:**

Supervisor consultation was positively associated with work engagement after adjusting for gender, age, education, income, and industry (*β* = 3.474; *p* < 0.001). The relationship remained significant after adjustment for perceived supervisor support, although the coefficient decreased (*β* = 1.315; *p* < 0.001).

**Conclusion:**

Supervisor consultation probably acted on work engagement in different ways than perceived supervisor support.

## 1 Introduction

Work engagement is defined as a positive, fulfilling, work-related state of mind, characterized by vigor, dedication, and absorption [1]. Workers with high work engagement are expected to have a high level of energy, mental resilience, and persistence even in difficult situations, and be strongly involved in and fully concentrated on their work [2]. The Job Demands–Resources (JDR) model suggests that work engagement mitigates the impact of demands at work such as burnout and psychological distress [3]. It therefore plays an important role in the psychological state of workers.

Supervisor support, defined as a supportive response from supervisors, including offering assistance and valuing employee contributions and wellbeing, contributes to work engagement [4]. Under the JDR model, supervisor support can be considered to be a job resource, a factor that stimulates growth, learning, and development within the organization [5]. Several previous studies have highlighted the importance of supervisor support in work engagement, psychological distress, and presenteeism [4,[6], [7], [8]]. Most of these studies were cross-sectional, except Mori et al. [9]. They also all measured supervisor support using the subjective views of employees.

In the workplace, some activities may be recognized as genuine examples of supervisor support. These include consulting with and providing feedback to employees to solve workplace-related problems, reward, or recognition from supervisors to employees for work tasks, and support from supervisors when employees have family problems. We believe that these activities are relatively common practice but not often recorded or measured, and no studies have assessed their impact on work performance.

To the best of our knowledge, no studies have examined the effect of supervisor consultation, as a practical element of supervisor support, on work engagement. We therefore wanted to examine the role of supervisor consultation on work engagement. We chose to adjust factors related to perceived supervisor support because it seemed likely that employees with high perceived supervisor support would consult their supervisors when they encountered a problem, and that this may influence the practice of supervisor consultation. We also chose to adjust other covariates, including age, gender, education, income, and industry, to justify their inclusion in the model. Our hypothesis was that the actual practice of supervisor consultation would positively influence work engagement even after adjustment for perceived supervisor support and covariates.

## 2 Materials and methods

### 2.1 Study design and participants

This was a prospective cohort study covering all regions of Japan. The study was conducted online in collaboration with a private data collection company. Only individuals registered with the company could respond to the survey. The protocol of this study has been published elsewhere [10]. The sample was stratified by workers' status, gender, age, and region to be consistent with the characteristics of the Japanese workforce [11]. Overall, 16,629 participants were matched for prospective cohort observation for a one-year period. Data were screened for working style. Participants who were self-employed (*n* = 1,423), worked in a family business (*n* = 250), or were company executives (*n* = 940) were removed from the sample because they had minimal chance to interact with a supervisor. This left a total of 14,026 participants for analysis. Data were collected from February 2022 to March 2023. The study was approved by the Ethics Committee of the University of Occupational and Environmental Health, Japan (R3-076) and complied with the Checklist for Reporting Results of Internet E-Surveys (CHERRIES) [12] to minimize potential bias during data collection.

### 2.2 Work engagement

Work engagement was measured one year after the first data collection on supervisor consultation. It was examined using the nine-item Japanese version of the Utrecht Work Engagement Scale (UWES-9) [13]. The nine items all used a Likert-type scale with answers from 0 (never) to 6 (always). Data were presented using a total score, with a minimum score of 0 and maximum score of 54.

### 2.3 Supervisor consultation

Supervisor consultation was measured using a single question “Do you actually consult with supervisor when you have problems about current work, work life or health problems?” Participants were asked to respond “yes” or “no.” The supervisor was defined as the person who supervises the work of the employee, and not by the system or culture, which usually reflects the definition of leadership or management. We did not specify the hierarchical position of the supervisor, although we considered that most participants would refer to their direct supervisor when answering the question.

### 2.4 Covariates

The covariates were age, gender, education, income, industry, and perceived supervisor support. Age was numerical, based on data from the online questionnaire. Gender was self-reported and categorized as male or female. Education level was identified by the most recent school from which the participant had graduated and categorized into low (junior high school and high school), medium (vocational school and junior college/technical college), and high (university and graduate school). Annual income was grouped into six categories: less than 4 million yen, 4–6 million yen, 6–8 million yen, 8–10 million yen, 10–12 million yen, and more than 12 million yen. For industry, participants were given 20 options and were asked to select the option that was closest to their employer. The options were “agriculture, forestry; fishery”; “mining, quarrying and gravel extraction”; construction; manufacturing; electricity, “gas, heat and water supply”; information and communication; transportation and postal; wholesale and retail; finance and insurance; real estate and goods leasing business; “academic, research and professional services”; accommodation and food services; life-related services and entertainment; education and learning support; medical and welfare; composite service business; service not classified elsewhere; public service not classified elsewhere; and unclassified industry. Perceived supervisor support was measured using a single question: “Does your supervisor support employees to work actively and lead a healthy life?” The answer options were “yes,” “reasonably often,” “not really,” and “not at all.”

### 2.5 Statistical analysis

We examined the distribution of work engagement and covariates, by supervisor consultation category. We then used linear regression analysis to examine the association between supervisor consultation and work engagement. Model one adjusted for age, gender, education, income, and industry. Model two additionally adjusted for perceived supervisor support. The significance value was set at *p* < 0.05. All the statistical analyses used Statistical Program for Social Science, Faculty Packs version 29 (IBM, Armonk, NY, USA).

## 3 Results

The majority of the participants had graduated from university or graduate school (52.4%) and had an annual income of eight million yen or less (70.4%). The biggest groups worked in manufacturing (17.8%), medical & welfare (14.3%), or wholesale & retail (10.3%). Details are shown in Table 1.

**Table 1:** Distribution of covariates among supervisor consultation group

	Supervisor consultation		Total
	No	Yes	
*N*	10,896	3,130	14,026
Sex, *n* (%)			
Male	5,940 (54.5%)	1,756 (56.1%)	7,696 (54.9%)
Female	4,946 (45.5%)	1,374 (43.9%)	6,330 (45.1%)
Age, mean (SD)	47.20 (12.86)	44.45 (12.63)	46.59 (12.86)
Education, *n* (%)			
Low	2,820 (25.9%)	732 (23.2%)	3,552 (25.3%)
Medium	2,397 (22%)	729 (23.1%)	3,126 (22.3%)
High	5,679 (52.1%)	1,669 (53.7%)	7,348 (52.4%)
Income, *n* (%)			
Less than 4 million Yen	2,847 (26.1%)	664 (21.2%)	3,511 (25%)
4–6 million Yen	2,724 (25%)	776 (24.8%)	3,500 (25%)
6–8 million Yen	2,195 (20.1%)	668 (21.3%)	2,863 (20.4%)
8–10 million Yen	1,480 (13.6%)	499 (15.9%)	1,979 (14.1%)
10–12 million Yen	683 (6.3%)	227 (7.3%)	910 (6.5%)
more than 12 million Yen	967 (8.9%)	296 (9.5%)	1,263 (9%)
Industry, *n* (%)			
Agriculture & Forestry	58 (0.5%)	12 (0.4%)	70 (0.5%)
Fishery	6 (0.1%)	2 (0.1%)	8 (0.1%)
Mining, quarrying & gravel extraction	6 (0.1%)	7 (0.2%)	13 (0.1%)
Construction	478 (4.4%)	144 (4.6%)	622 (4.4%)
Manufacturing	1,942 (17.8%)	548 (17.5%)	2,490 (17.8%)
Electricity, gas, heat & water supply	147 (1.3%)	41 (1.3%)	188 (1.3%)
Information & communication	523 (4.8%)	166 (5.3%)	689 (4.9%)
Transportation & postal	548 (5%)	144 (4.6%)	692 (4.9%)
Wholesale & retail	1,139 (10.5%)	305 (9.7%)	1,444 (10.3%)
Finance & insurance	503 (4.6%)	127 (4.1%)	630 (4.5%)
Real estate & goods leasing business	230 (2.1%)	50 (1.6%)	280 (2%)
Academic, research & professional services	258 (2.4%)	64 (2%)	322 (2.3%)
Accommodation & food services	300 (2.8%)	110 (3.5%)	410 (2.9%)
Life-related services & entertainment	250 (2.3%)	68 (2.2%)	318 (2.3%)
Education & learning support	703 (6.5%)	214 (6.8%)	917 (6.5%)
Medical & welfare	1,443 (13.2%)	564 (18%)	2,007 (14.3%)
Composite service business	111 (1%)	30 (1%)	141 (1%)
Service not classified elsewhere	1,090 (10%)	277 (8.8%)	1,367 (9.7%)
Public servant except elsewhere classified	753 (6.9%)	193 (6.2%)	946 (6.7%)
Unclassified industry	408 (3.7%)	64 (2%)	472 (3.4%)
PSS score, mean (SD)	16.05 (4.39)	19.16 (3.31)	16.74 (4.38)

PSS, Perceived Supervisor Support.

Linear regression analysis showed that supervisor consultation was positively associated with work engagement (*β* = 3.113; *p* < 0.001 in the crude analysis and *β* = 3.474; *p* < 0.001 in model one). When the model was adjusted for perceived supervisor support in model two, the coefficient was reduced but the association remained significant (*β* = 1.315; *p* < 0.001). Details are shown in Table 2.

**Table 2:** Linear regression analysis of supervisor consultation toward work engagement

Variables	Crude			Model 1			Model 2		
	Coefficient∗	SE	*p*	Coefficient∗	SE	*p*	Coefficient∗	SE	*p*
Constant	22.107	0.102	<0.001	8.822	0.569	<0.001	1.615	0.6	<0.001
Supervisor consultation	3.113	0.215	<0.001	3.474	0.210	<0.001	1.315	0.216	<0.001

Model 1: adjusted for sex, age, education, income, and industry.

Model 2: adjusted for model 1 and PSS score.

∗ Unstandardized coefficient.

## 4 Discussion

We found that supervisor consultation, as an actual practice demonstrating supervisor support, had a positive association with work engagement over a one-year period. The relationship weakened when adjusted for perceived supervisor support, but a significant correlation was maintained. Adjustment for perceived supervisor support is important to differentiate actual supervisor support through consultation from other support that might be more indirect, such as through creating policies and regulations. This approach differed from other studies that mostly used perceived support to measure supervisor support. Our results suggest that supervisor consultation may act on work engagement through different pathways. This in turn means that both supervisor consultation and perceived supervisor support are probably needed to maintain work engagement.

Consultation is a process of communication used to get advice or obtain opinions [14]. In companies, it is also a process by which management and employees jointly examine and discuss issues of mutual concern [15]. In this case, the supervisor is acting as a representative of the management. One important issue for consultation is employees' problems. In this study, we defined consultation between supervisor and employee as discussion of employee problems related to current work, work life, or health.

Previous work has suggested that if consultation is maintained well between management and employees, it will have a positive impact among employees, including greater trust in the management, improved employee performance and satisfaction, and better employee engagement [16]. Supervisor consultation sessions might also include specific processes such as coaching and performance feedback. Rofcasin et al. found that both coaching and performance feedback positively contributed toward work engagement [17]. They suggested that a good consultation process, carried out by a supervisor as a representative of the management, will probably support work engagement. Drawing on the JDR model, supervisor consultation may therefore act as a job resource supporting work engagement, like perceived supervisor support [5].

Our study emphasized the importance of actual supervisor support. Its effect on work engagement remained significant even when the relationship was adjusted for perceived supervisor support. This highlighted that supervisor consultation, as a direct contact communication channel, is important to maintain work engagement. This real action of supervisors probably affects work engagement differently from a subjective feeling among employees that they are likely to be supported by their supervisor. The latter is more hypothetical, and possibly indirect.

Implementation of good consultation processes between supervisors and employees needs careful thought. There are aspects that should be put in place or maintained, such as the existence of policy, arrangement of method and consultation process, training for supervisors, and monitoring of the policy's implementation [16]. One example of a possible method is counseling. This is an important role for supervisors that may be carried out through a consultation process [18]. Company efforts such as creating channels for counseling are important to make sure that supervisor consultation is implemented effectively.

Our study had some limitations. We used a self-reported questionnaire to collect the data and did not measure the frequency of consultation between supervisor and employee or identify the effect on work performance. Measurement of frequency of consultation would provide more detailed analysis about the effect of the number of sessions on work engagement. We also used terminology about the “last school” from which participants had graduated to define education level. This may have led to misunderstandings if participants participated in vocational training after their formal education. Future research should measure frequency of consultation, define the level of education as the highest level of education from which participants had graduated, rather than the most recent, and provide a measure of work performance.

Despite these limitations, our study provides useful evidence that real action taken to demonstrate supervisor support, in the form of supervisor consultation, has a positive impact on work engagement across a range of workplaces. It also suggested that active consultation should be combined with actions to increase perceived support from supervisors to maximize the impact on work engagement.

## Conflicts of interest

N/A.

## Author contributions

NA, TN, KO and KM, study design; TN, KO, MN and KM, data collection; NA and TN, data analysis; NA, draft of manuscript; All authors have reviewed, edited, and approved the final manuscript.
